# Hemianopia and Features of Bálint Syndrome following Occipital Lobe Hemorrhage: Identification and Patient Understanding Have Aided Functional Improvement Years after Onset

**DOI:** 10.1155/2019/3864572

**Published:** 2019-03-25

**Authors:** Nicola McDowell, Gordon N. Dutton

**Affiliations:** ^1^Institute of Education, Massey University, Albany Drive, Palmerston North 4474, New Zealand; ^2^Department of Vision Sciences, Glasgow Caledonian University, Cowcaddens Road, Glasgow G4 0BA, UK

## Abstract

*Introduction. *Cerebral visual impairment (CVI) can present around birth or any time thereafter. Homonymous hemianopia is a common feature. The concept that functional improvement is unattainable augurs against active management. Dorsal stream dysfunction (or Bálint syndrome when severe) results from bilateral posterior parietal dysfunction but may go undetected, especially in children.* Case Presentation. *At 16 the patient suffered spontaneous left occipital lobe brain hemorrhage from a ruptured arteriovenous malformation. This was surgically excised. Short lived right upper limb intermittent jerking, with additional left sided weakness, ensued. Anomalous EEG recordings, with right-sided bias, arose from the posterior temporoparietal area. A right homonymous hemianopia was evident. During the ensuing 17 years she experienced multiple complex difficulties, until, at a lecture describing how to identify and support children with CVI, she realized she herself had many of the difficulties described. Visual assessment identified hemianopia and dorsal stream dysfunction.* Discussion.* Following identification, characterization, and explanation of the impact of her visual difficulties, she both gained greater awareness of her visual difficulties and their impact and developed a range of strategies leading to functional improvement of her visual field loss and amelioration of her dorsal stream dysfunction, with great improvement in quality of life.

## 1. Introduction

Cerebral visual impairment (CVI) can present around birth, often in relation to prematurity [1], hypoxic ischemic encephalopathy [2], and neonatal hypoglycemia [3], or it can be acquired at any time thereafter. Acquired causes include closed head injury, epilepsy, metabolic disorders, CNS infection [2], and brain neoplasia [4]. The most frequent manifestation of acquired CVI is homonymous hemianopia [5–7]. However, this common impairment often goes uncharacterized and untreated, owing to the belief that functional recovery from visual field loss is unattainable [6], despite recognition of the potential for persisting hemianopic blindsight, comprising the facility to respond predictably to visual stimuli in the hemianopic visual field, but without visual consciousness [6, 8, 9].

The identification of blindsight may facilitate visual rehabilitation in patients with homonymous hemianopia [10], founded on the hypothesis that residual nonconscious visual capacities can potentially be rendered conscious through training [6]. The requisite methodology has been shown to be beneficial in the case described by Arcaro and Thaler [11] of a patient who lost vision due to bilateral occipital infarction. Two years after the injury, the patient developed the Riddoch phenomenon enabling her to walk around obstacles and to mirror other's movements, despite reporting that she had no useful vision [11, 12]. The patient was encouraged and empowered to develop strategies to use motion and self-motion to improve her visual abilities and her confidence to trust them. After an initial period of using these approaches to aid her vision, she became able to relinquish them as she learned to move freely through cluttered environments and achieve actions such as catching a moving ball [11].

Another well described CVI that results from cerebral injury in adults is Bálint syndrome [13]. This is caused by bilateral posterior parietal lobe dysfunction or injury [14]. It is characterized by three specific visual difficulties that impact on a person's visual access to the spatial characteristics of their surroundings. These comprise the following: simultanagnosia (an inability to see more than one or two objects at the same time; (described by Grüsser and Landis [4] as a ‘disturbance of grasping the whole'), optic ataxia (impaired visual guidance of movement) [15] psychic paralysis, or apraxia of gaze (inability to volitionally direct visual gaze despite the requisite motor substrate) [13, 16]. Bálint syndrome has been infrequently reported in children, but recent case studies have highlighted that children can be significantly impacted by this triad of visual spatial difficulties [16].

Bálint syndrome-like features can occur in patients who have experienced bilateral posterior parietal lobe injury, but who are not as severely affected as those with Bálint syndrome in its most severe form [17]. This lesser, but yet still complex form of Bálint syndrome, is referred to as dorsal stream dysfunction and commonly occurs in children [18]. The dorsal visual stream runs within the superior longitudinal fasciculus from the occipital lobe to the posterior parietal cortex and is often referred to as the “vision for action” or the “where” pathway [19]. Difficulties due to dorsal stream dysfunction include the following:

- avoidance of crowded and cluttered environments or the increase of negative behaviors when in such places, such as tantrums and crying [20],
- social withdrawal due to difficulty finding friends in groups of people [21],
- avoidance of schoolwork due to difficulty copying information, difficulty simultaneously processing incoming visual and auditory information, and impaired ability to find numbers on a printed page and problems locating items, both on their work station and within the wider classroom environment [21, 22],
- reluctance to move around both familiar and unfamiliar environments due to past embarrassing experiences of walking into objects and people and appearing clumsy [22],
- an unwillingness to participate in sporting activities due to issues with depth perception and eye-hand coordination [22],
- memory difficulties, as visual information cannot be encoded in a robust and clear way [23].

We report a patient with combined hemianopia and dorsal stream dysfunction, for whom the insights gained from identification and explanation have changed her life for the better.

## 2. Case Presentation

**Figure 1:**
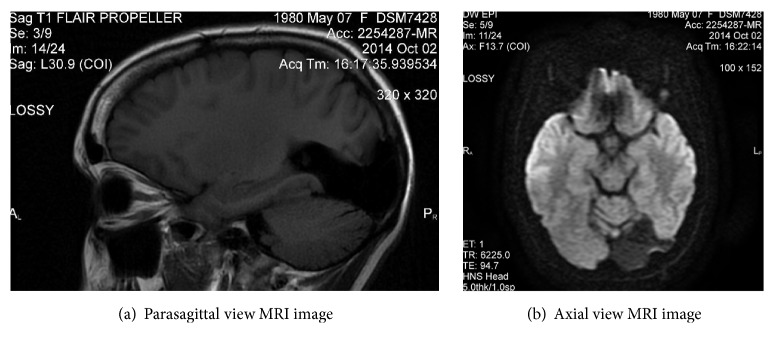
(a) Parasagittal and (b) axial T2 weighted MRI images of the patient's brain after removal of her left occipital arteriovenous malformation.

A 16-year-old left handed girl suffered a spontaneous brain hemorrhage into her left occipital lobe. She lost consciousness and was resuscitated from respiratory arrest. Imaging showed left occipital and left frontoparietal subdural hematomata. Drug induced coma was implemented and maintained for eight days prior to left parietooccipital craniotomy and excision of the causative left occipital arteriovenous malformation (Figures 1(a) and 1(b)). At surgery small arterial feeders were evident 5cm from the midline. The hematoma cavity was evacuated. The arteriovenous malformation extended into the lateral horn of the left lateral ventricle and across the surface of the tentorium, draining into the transverse sinus. The medial aspect of the left occipital lobe was disconnected from the midline and the affected 4 x 4cm area was hinged up onto the draining vein. The vascular pedicles were coagulated and divided, and the vascular malformation was removed. Shortly following the surgery, the patient displayed intermittent jerking of the right upper limb, as well as marked weakness of the left side. EEG testing revealed irregular brain waves in the posterior temporal and parietal regions with a right-sided bias. Both the jerking and the weakness resolved over 4 days. A right-sided homonymous hemianopia became evident. On discharge, the patient and her parents were informed of a possibility of spontaneous recovery of the hemianopia, but this was deemed unlikely. Her neuropsychological tests showed focal visual dysfunction, suggestive of difficulties in visual search (Table 1), but their origin was not identified at the time. For the following 17 years, the patient experienced challenges with many aspects of her life, including her mobility, her ability to access learning material at school, her social interactions, specific daily living activities including finding items on a supermarket shelf or clothes in her wardrobe, and the control of emotion. In 2013, at a conference presentation on identifying and supporting children with CVI, the patient recognized the difficulties described as being identical to her own. The patient introduced herself to the presenter and explained this.

Visual assessment confirmed persisting right hemianopia. No evidence of visual impairment in the sighted visual field [24] was elicited. A body turn to the right (without a head turn) slightly extended the field of vision to the right, suggestive of an additional element of right inattention. Nevertheless, repeated small discreet finger movements made by the examiner in multiple locations within the hemianopic field were correctly intuited on more than 70% of occasions, consistent with blindsight. Uncorrected visual acuities and stereopsis were in the normal range. Structured history taking for evidence of perceptual visual impairment [25] revealed great difficulty finding an item from within clutter or within a pattern and great difficulty copying, consistent with simultanagnostic vision. Standard eye movement examination was normal, with normal convergence, pursuits, and saccades to command (e.g., ‘look right'), but instruction to look at specific items nominated by the examiner resulted in anarchic visual search movements until the specified item was eventually found. This feature is consistent with apraxia of gaze, a phenomenon known to accompany simultanagnostic vision [17]. When asked to look at a large group of people, the patient could only identify the presence of one person. Careful observation of eye movements during such search consistently showed them to be sparse and apparently random. In an outdoor café she was asked to count the number of support poles for the sun-shading roof. She randomly found and identified 3 out of 11, with the same lack of visual search strategy.

The accuracy of the patient's visual guidance of reach was evaluated. Careful observation of the in-flight gap between the fingers, and their orientation while reaching for discreet targets in the intact visual field showed consistent features of a gap much wider than necessary, with inaccuracy of orientation [19]. These features are consistent with a degree of accompanying optic ataxia.

The patient was given a detailed tutorial concerning the nature of her vision and strategies that she could employ to make best use of her vision.

## 3. Discussion

Receiving an in-depth explanation for the unexplained challenges that she had lived with for 17 years had an immediate impact on the patient's feelings of self-worth and overall well-being. Just the knowledge that she had been living with a significant visual impairment that had only recently been identified helped her to understand that it was this impairment that impacted on many aspects of her daily life, as opposed to her feeling ‘useless' and unable to achieve simple activities, such as the grocery shopping for her family.

As each specific visual difficulty was assessed and thoroughly explained to her, she found herself able to connect her anomalies of visual function, to the specific challenges she experienced. For example, the reason she did not like spending time in crowded and cluttered environments (supermarkets and shopping malls) was not only due to her hemianopia, but also because of a combination of her simultanagnostic visual dysfunction and hemianopia together. Also, the reason she often got frightened by people suddenly ‘looming' in front of her, when walking in a busy environment, was because of a combination of the hemianopia and her impaired visually guided movement making it difficult for her to judge how far away people actually were. But probably the most important concept that she learnt about her CVI was that she had the potential to improve her visual functioning through personal endeavor.

As with patient MC, described by Arcaro and Thaler [11], the patient was surprised that she had been able to accurately respond to stimuli in her right visual field, even though she felt she could not ‘see' any image. Although she had previously been aware that at times she was able to detect movement in her right visual field, the concept of blindsight had never been explained to her. Therefore, her new recognition that she had the potential to improve her awareness of movement within her right visual field, to begin to look and see, was empowering [26]. As with MC, the patient was encouraged to develop strategies to use motion and self-motion to improve her visual abilities and her confidence to trust them [11]. Although at first she used the motion of head nodding to experiment with detecting visual stimuli on her right in her home environment, it was not a strategy she used when out and about. However, not long after receiving her CVI diagnosis following the conference presentation, she began to become more aware of movements in her right visual field. When this occurred, she made a conscious effort to try and interpret what she was seeing, instead of ignoring it, as she had in the past. She soon found that the more conscious awareness she gave to understanding what she was seeing, the more she became able to interpret her visual responses to the movements on her right. This is in line with studies focusing on increasing the detection and awareness of stimuli within the blind hemifield, which have shown that with repeated stimulation, the incidence of reported awareness also increases [27] and may well relate to the type of increased saccadic amplitudes resulting from more structured training in visual search, recently shown to be effective in extending the functional field of vision in children with hemianopia [28].

Each day she challenged herself to interpret movement she was detecting on her right side. Her approaches included attempting to detect any movement the driver on her right was making while she was sitting in the left hand passenger seat, looking straight ahead; walking on the left hand side of the path when out for her daily walks and trying to establish the exact moment, runners, other walkers, or cyclists passing her on her right hand side (while wearing headphones and listening to music so she could not hear them); and choosing a place to sit in her lounge that meant that the doorway was on her right and trying to detect any movement through the door while looking straight ahead at the television while ensuring she acknowledged and described everything she saw when detecting stimuli on her right side.

These approaches are in line with visual field training techniques described by Pollock and Hazelton [29] where patients are trained to detect stimuli that are repeatedly presented in their blind hemifield, to help increase their overall sensitivity to them by helping the brain to enhance visual awareness. Within the first year of implementing these strategies, the patient was beginning to be aware of more stimuli in her right visual field. After two years of her continuous visual self-training regime, she was surprising even herself on the occasions that she detected and correctly identified that the family (black) cat was on her right side or that someone was walking past her from behind. Her most exciting detection was when she looked up from reading her book to respond to an air hostess at the exact moment she appeared on her right side. With the continued self-training, over time she also began to detect more and more stimuli that were unrelated to her training regime, for instance, detecting changes in a familiar environment, such as furniture being moved.

**Figure 2:**
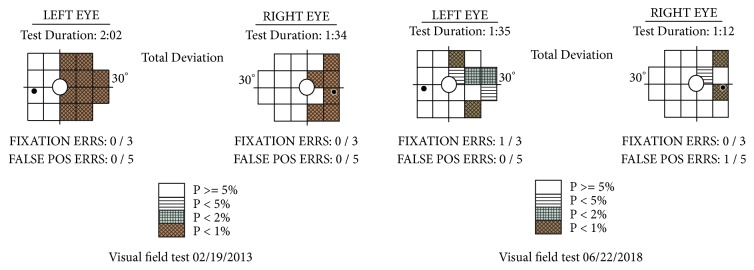
It comprises images of two visual field plots on a Humphrey FDT Viewfinder, demonstrating improvement in visual fields. The first image is from 02/19/13 and the second is from 06/22/18.

During the six years following her diagnosis, visual field testing was performed by her optometrist using the Humphrey FDT Viewfinder (Figure 2). The first was undertaken in 2013 and showed little change from the plots of 1996 following her brain hemorrhage. Successive testing, however, revealed progressive improvement in the right visual field of both eyes. This remarkable functional improvement supports the hypothesis that it is potentially possible for neuroplastic brain change to occur through focusing on the belief and expectation that things can change, while striving to gain greater conscious visual awareness, perhaps through modulating processes in the brain that control and facilitate change [30].

As well as focusing on functionally improving her visual field, the patient also concentrated on improving her overall visual function. An important aspect of this was to firstly develop an in-depth understanding of her Bálint-like symptoms / dorsal stream difficulties of simultanagnostic visual dysfunction, optic ataxia, and apraxia of gaze and how they impacted on her in everyday life. This meant focusing on each specific issue separately, followed by the combined impact of living with a significant visual spatial impairment. Once the patient understood the nuances of her impaired unconscious visual functions, she was able to compare her current experiences of the visual world to how she had previously seen the world prior to her brain hemorrhage. With this unique insight, she was able to develop strategies to help improve her visual functioning in challenging activities and environments.

As a dorsal stream dysfunction largely affects nonconscious visual functions [19], the patient used the analogous approach to the one she used for developing her Riddoch phenomenon and focused on becoming more conscious of what was occurring when she was experiencing difficulties as a result of her simultanagnostic vision, optic ataxia, and apraxia of gaze. To strive to develop effective conscious viewing, she followed the concept outlined by Doidge [31] of using conscious actions to overcome unconscious processes, which requires a focused meditative level of concentration. This process has been used successfully for a man suffering from Parkinsonian symptoms, also described by Doidge [31] as using a form of conscious walking, whereby he trained himself to overcome many of his symptoms, by forcing himself to focus on the specific actions of walking and not letting his body adopt compensatory movements as a result of his Parkinson's disease.

At first, she adopted a different form of conscious viewing for each of her visual difficulties. For instance, for her simultanagnostic vision, she developed a strategy that she termed as ‘wagon wheel visual fixation.' This strategy required the patient to consciously recognize when her gaze had wandered from what she was looking at in a crowded or complex environment, to enable her to build up the complete visual scene over time. For this strategy to be effective, she found the first step was to establish an anchor point (an obvious and appealing visual target in the middle of the chosen environment). She then trained herself to consciously recognize whenever her gaze had moved from this anchor point and work hard to force her gaze back to it. This process was repeated over and over again, applying a wagon wheel approach to consciously chosen eye movement, until the whole scene had been mapped out.

To aid her mobility in relation to her optic ataxia, especially in busy environments, she again adopted a process of conscious viewing and worked hard to focus her attention on processing and interpreting the visual information in her surroundings. For this, as she was moving, she focused her visual attention upon the space ahead of her that she would be moving in to. She then repeatedly glanced at the different elements in her travel pathway to make sure she had an enhanced opportunity to accurately map the environment around her. To ensure that this process was effective, she had to mentally block out all other sensory distractions, so that she could focus solely on processing the visual information.

Both of these techniques were difficult to implement and although she could immediately see the potential benefits of each strategy, they both had to be repeatedly practiced in the environments that required such strategies, before she was confident with her ability to implement them. However, while experimenting with these strategies, the patient also realized that she could only use them when she was feeling calm and relaxed. If anxious or stressed in any way, especially in a complex environment, she found them difficult to implement. Although she had developed a very good understanding of what types of environments were difficult for her (supermarkets, busy restaurants, shopping malls, and crowded parks), this knowledge alone was insufficient to help her cope in such locations. She then came to realize that along with her unconscious visual behaviors, she was also experiencing unconscious emotional reactions, including anxiety, panic, and high levels of stress as a result of the dorsal stream dysfunction [32].

The difficulty in implementing strategies requiring forced conscious actions necessitating intense cognitive functioning is in line with the awareness that when a person's sympathetic nervous system causes a fight or flight response in their primitive brain, their ability to think logically and coherently is absent [33]. The patient, therefore, realized that to be able to implement the specific visual strategies she had developed, she needed to identify techniques to keep herself calm, to allow any chance of overcoming both her visual difficulties and her behaviors due to her emotional reactions. To help reduce her constant high levels of anxiety when in complex environments, the patient began daily mindfulness practice, which has been shown to be useful in preventing feelings of anxiety and stress by helping a person to concentrate on their breathing and to not focus on negative or disruptive thoughts or feelings [34]. To aid in her practice, she used an online mindfulness based intervention program, as this has been proven to be effective in treating a variety of mental health conditions in adults, including anxiety and stress [35–38]. Although there is currently no literature concerning the use of cognitive based therapies such as mindfulness to support children and adults with CVI, they have been shown to be effective in helping children with High Functioning Autism Spectrum Disorder (HFASD) and anxiety disorders [39]. The concept of focusing on her breathing also helped reinforce the strategy of focusing on an anchor point when implementing the wagon wheel visual fixation process. Over time, focusing on an anchor point became easier to achieve, which supports the concept that regular mindfulness practice changes the brain patterns that cause day to day anxiety and stress [34].

With regular practice of both the mindfulness exercises to reduce environment based anxiety and her specific visual strategies of wagon wheel visual fixation and conscious viewing, the patient started to notice that the uncomfortable compensatory behaviors she had developed on account of her visual difficulties started to diminish. For instance, she stopped withdrawing from social activities and interactions and instead started engaging in activities such as going out for dinner with friends once more. She also started feeling more confident in her mobility, even in crowded environments, and felt more comfortable travelling independently in these environments. She found it easier to control her emotions and greatly reduced the number of occasions where she experienced high levels of anxiety and stress around her visual difficulties. It also helped to improve her overall well-being and confidence and helped to render normal daily activities, such as taking her children to the playground less fatiguing and more enjoyable.

Most significantly, she also noticed that her visual functioning had greatly improved and that she was less handicapped by her simultanagnostic vision, optic ataxia, and apraxia of gaze. She could now cope in busy, crowded environments for longer periods of time. She did not feel as clumsy and was not frightened by people looming in front of her. She also found it easier to spot objects in the distance that were pointed out to her, and she felt confident in her ability to handle any situation and environment that she found herself in.

## 4. Conclusions

What this case highlights is that for patients who do not have other cognitive disorders (such as memory or frontal disorders), it is possible to overcome some of the most disabling effects of a homonymous hemianopia and dorsal stream dysfunction. This is despite the fact that both these conditions can greatly impact on the quality of life for those affected, both physically and mentally [27, 29, 32]. However, for this to happen, the patient needs to firstly, whenever possible, fully understand the nature of their visual difficulties and how they impact on their day-to-day living. This requires timely and accurate diagnosis of not only the overarching condition of CVI, but also the specific visual difficulties of simultanagnostic vision, optic ataxia, and apraxia of gaze. In regard to their hemianopia, patients also need to be introduced to the concept of blindsight and the Riddoch phenomenon, so that if they do experience subconscious or conscious perception of movement in their blind visual fields, they are aware that this could aid their rehabilitation. Secondly, the patient also needs to be empowered through this understanding and the awareness that they have the ability to possibly functionally improve their impairments, through utilizing the resilience and plasticity of the brain to be able to reorganize itself after brain damage [27, 29, 40]. This case study also gives weight to the hypothesis that along with passive bottom up processes that can influence the cortical reorganization, neuroplastic brain change can occur through simply the belief and expectation that things can change [30], if the patient has the desire to implement strategies to bring about such change.

## Figures and Tables

**Table 1:** Neuropsychology tests as reported (dates of assessments: A. 30.9.96, B. 16.1.1998).

*Intelligence *Wechsler Adult Intelligence Scale – Revised (WAIS -R)
(Pre-accident: High average range)
A. Not done
B. Superior range of functioning
*Reading *Wide Range Achievement – Third Ed (WRAT-3) and SCOLP spot the Word subtest
A. Low average (reading single words out loud)
B. Slight improvement into the average range (reading single words)
*Confrontation naming *Boston Naming Test
A. Low average range
B. Average range*∗*
*Speed of information processing *SCOLP speed of compression subtest
A. 10^th^ centile
B. Improvement into the average range*∗*
*Attentional and psychomotor skills *WAIS-R Digit Symbol subtest and Trial Marking Test
A. Speed of visual motor responding and visual scanning low average
B. Slight improvement into the average range*∗*
*Verbal memory abilities *California verbal learning test
A. Intact abilities in learning new verbal material preserved abilities in reading recently learned verbal material from long-term memory
B. ‘No change' reported
*Visuospatial skills and visual memory abilities *Rey complex figure test
A. “Misplacement of detail”
B. “Copied the design in piecemeal fashion as she was unable to see the entire figure at a glance”
*Executive and problem-solving abilities *Trail making test
A. Slow speed of information processing
B. Now has no problems alternating her attention between number and letter concepts under time pressure
*Cognitive flexibility and problem-solving skills *Wisconsin card sorting test (for conceptual flexibility, hypothesis formation, testing abilities,
skills in using feedback to modify problem-solving)
A. Above average range
B. Above average range

*∗* ‘Inconsistent with WAIS-R test results'

The items underlined are indicative of persistent difficulties with visual information processing.
